# The mediating role of perceived emotional intelligence: examining the impact of affective job satisfaction on organizational identification among Chinese technological workers

**DOI:** 10.3389/fpsyg.2024.1285853

**Published:** 2024-08-29

**Authors:** Yuege Lai, Ge Gao, Baiyan Du

**Affiliations:** ^1^College of Teacher Education, Quzhou University, Zhejiang, China; ^2^College of International, Zhengzhou University, Henan, China

**Keywords:** emotional intelligence, mediation, affective job satisfaction, organizational identification, organization

## Abstract

**Introduction:**

Breaking new ground in the exploration of workplace dynamics, this study pioneers an investigation into the mediating role of perceived emotional intelligence (PEI) in the relationship between affective job satisfaction and organizational identification among Chinese technological workers. This novel focus addresses a critical gap in existing research, particularly in understanding the psychological underpinnings within this specific cultural and professional context.

**Methods:**

Involving 392 workers aged 23 to 60, our research offers a comprehensive examination of how the three subdimensions of PEI - attention, clarity, and emotional repair - interact with job satisfaction to influence organizational identification. Through extensive questionnaires, we assessed these subdimensions alongside affective job satisfaction and the workers’ identification with their organization.

**Results:**

Our findings reveal a significant, positive correlation between job satisfaction and all PEI subdimensions. Notably, while emotional clarity and emotional repair showed a positive relationship with organizational identity, attention did not. Further analysis highlighted the substantial direct impact of Affective Job Satisfaction on Organizational Identification, with emotional clarity and emotional repair playing critical mediating roles.

**Discussion:**

These insights illuminate the unique function of Perceived Emotional Intelligence as a mediator and enhancer in the relationship between job satisfaction and organizational commitment. The results underscore the necessity of integrating strategies to cultivate emotional intelligence in the workplace, potentially leading to stronger organizational ties and improved overall worker well-being. By shedding light on these complex psychological mechanisms, our study not only enriches the theoretical landscape but also offers practical guidance for fostering healthier, more productive work environments.

## Introduction

Currently, it is increasingly evident how emotions influence our lives, and the importance of possessing adequate emotional intelligence to confront challenges in various social contexts (Molero-Jurado et al., 2021; Molero Jurado et al., 2022). Salovey and Mayer (1990) Model of Emotional Intelligence, a widely supported framework, defines Emotional Intelligence as a form of social intelligence that empowers us to recognize and understand our own emotions and those of others, thereby guiding our actions and thoughts.

Over decades, numerous research studies have been conducted in different areas such as adulthood (Walker et al., 2022), health (Martins et al., 2010), romantic relationships (Jardine et al., 2022), and the workplace, among others (Miao et al., 2017). Particularly in Organizational Psychology, Emotional Intelligence is recognized for its positive influence on desirable outcomes like organizational commitment, prosocial behaviors, and job performance, and its potential to mitigate negatives such as stress and counterproductive behaviors (Miao et al., 2017, 2018).

Despite the abundance of research on this topic, the relationship between Emotional Intelligence in the workplace, Affective Job Satisfaction, and Organizational Identification has not been explored in detail. This lacuna is significant as understanding this relationship can offer insights into how employees’ emotional competencies contribute to their satisfaction and sense of belonging within an organization, which are critical factors for employee retention and organizational success. Therefore, this research aims to analyze the mediating role of Emotional Intelligence—specifically, emotional attention, clarity, and reparation—in the connection between Affective Job Satisfaction and Organizational Identification. By elucidating these dynamics, our study promises to enhance the understanding of how Emotional Intelligence can be leveraged to foster a more harmonious and productive workplace, particularly in sectors where emotional demands are high.

Building upon this foundation, our study ventures into relatively uncharted territory by focusing on the Chinese technological sector, a context that has been notably underrepresented in existing literature. While the impact of Emotional Intelligence on various workplace outcomes has been extensively documented, its specific role as a mediator between Affective Job Satisfaction and Organizational Identification within this unique cultural and professional milieu has not been thoroughly explored. This gap in research is particularly significant given the distinct socio-economic and cultural dynamics influencing the Chinese tech industry. Our study aims to fill this void by providing empirical insights into how Emotional Intelligence, particularly in terms of emotional attention, clarity, and reparation, influences the relationship between job satisfaction and organizational identification among Chinese technological workers. This novel approach not only contributes to the broader understanding of Emotional Intelligence in diverse professional contexts but also offers valuable implications for organizational practices in a rapidly evolving global technological landscape.

## Theoretical framework

The theories of intelligence proposed in the first half of the 20th century set the stage for the development of the first formal definition and model of Emotional Intelligence by Salovey and Mayer (1990). They defined Emotional Intelligence as a type of social intelligence that involves monitoring one’s own and others’ emotions, discriminating between them, and using this information to guide one’s thoughts and actions. Emotional Intelligence is organized into four interrelated and hierarchically structured areas: (a) Perception, appraisal, and expression of emotions; (b) Emotional facilitation of thought; (c) Understanding and analyzing emotions; and (d) Reflective regulation of emotions (Mayer et al., 2001).

The perception, appraisal, and expression of emotions refer to a person’s ability to accurately identify their own and others’ emotions and distinguish between appropriate and inappropriate expressions. This includes recognizing emotional content through voice tone, facial expressions, body language, etc. Individuals who respond effectively to their own emotions are also better at recognizing and responding to the emotions of others, indicating higher emotional intelligence (Mayer et al., 2001). Emotional facilitation of thought highlights the relevance of emotions in cognitive processes and how they direct attention toward salient information. This involves controlling how emotions are processed by our cognitive system and how they influence our thoughts. For instance, when a person is happy or sad, their thoughts tend to become more positive or negative, respectively (Salovey and Birnbaum, 1989).

Understanding emotions refers to the knowledge a person possesses about the emotional system, how emotions are cognitively processed, and how emotional information impacts comprehension and reasoning (George et al., 2022). Emotionally intelligent individuals recognize the characteristics of emotions within specific families, such as identifying fear and surprise as components of terror or faith and optimism as components of hope (Mayer et al., 2001). This dimension enables people to interpret complex emotions, recognize transitions between emotional states, and understand that they can experience multiple and contradictory feelings simultaneously (Sha et al., 2022).

Emotional regulation involves the capacity to be receptive to positive and negative emotional states and to reflect on these emotions to determine their usefulness, without suppressing or exaggerating them. It includes the skill of regulating both one’s own and others’ emotions. Emotional regulation prevents uncontrolled emotional responses in situations of anger, provocation, or fear. It allows individuals to perceive and experience their emotions without compromising their rational thinking (Valente and Lourenço, 2022). Moreover, emotional regulation facilitates complex emotional processes, promoting conscious regulation of emotions for intellectual and emotional growth (Arteaga-Cedeño et al., 2022).

To assess Emotional Intelligence, the Trait Meta-Mood Scale self-report measure is used. This scale consists of three dimensions (emotional attention, emotional clarity, and emotional repair) that assess a person’s meta-awareness of their emotional abilities, rather than their actual levels of Emotional Intelligence. Thus, some researchers (Salovey et al., 2002) suggest that this assessment focuses on Perceived Emotional Intelligence.

Numerous studies have explored the relationship between Perceived Emotional Intelligence and various constructs. For instance, Gohm et al. (2001) investigated how emotions influenced cognitive responses to stress among firefighters. They found that individuals with higher levels of emotional clarity exhibited more appropriate cognitive responses. In other research, Perceived Emotional Intelligence has been used to examine the quality of interpersonal relationships at work. These studies demonstrate that higher levels of Perceived Emotional Intelligence are associated with positive relationships with colleagues and supervisors, as well as higher job satisfaction (Gohm and Clore, 2002).

### Perceived emotional intelligence in the organization

Perceived Emotional Intelligence has gained interest in the organizational context as a predictor of important outcomes for individuals and companies. Different theoretical perspectives have emerged, influencing academic and organizational fields. In the organizational context, the significance of emotions in work life and their impact on organizational effectiveness, leadership, and team performance has been acknowledged (Zeidner et al., 2002).

Numerous research studies confirm that Perceived Emotional Intelligence contributes to better performance, improved relationships, and higher job satisfaction, benefiting both individuals and organizations (Hopkins and Bilimoria, 2008; Turner and Lloyd-Walker, 2008). These positive effects are not limited to leadership positions but also extend to intermediate roles, where improved results and higher-quality relationships with colleagues are achieved (Durra, 2023). In a study conducted with university professors and technical employees (Batista et al., 2022), Perceived Emotional Intelligence was found to influence job satisfaction in both groups. The study revealed that emotional skills are valuable resources for managing workplace demands, leading to similar effects on job satisfaction in both technical and academic roles. Kafetsios and Zampetakis (2008) found in their research with 523 primary and secondary school teachers that higher levels of Perceived Emotional Intelligence were associated with greater job satisfaction and predicted affect at work. Similarly, Mérida-López et al. (2020) found a direct relationship between Perceived Emotional Intelligence and work engagement, with higher Perceived Emotional Intelligence associated with greater commitment to work.

Meta-analyses further support the strong connections between Perceived Emotional Intelligence and job satisfaction. Humphrey (2015) showed that a leader’s emotional intelligence positively relates to subordinate job satisfaction at the individual and group levels (Humphrey, 2015). A recent meta-analysis also demonstrated that Perceived Emotional Intelligence is positively related to organizational commitment, organizational citizenship behavior, job satisfaction, and job performance, while negatively related to job stress (Doǧru, 2022).

### Affective job satisfaction

Affective job satisfaction is a positive, overarching emotional response toward one’s job as a whole (Moorman, 1993). Often synonymous with general or global satisfaction, affective job satisfaction is assessed through items asking individuals how much they enjoy their work. In contrast, the evaluation of the cognitive facets of job satisfaction emerges from a rational comparison of job conditions against a desired, expected, or promised standard (Moorman, 1993). Thus, it becomes evident that affective job satisfaction and cognitive satisfaction are distinct constructs, albeit interrelated to some extent. Numerous empirical studies have underscored the connection between job satisfaction and emotions. Weiss argued that usual assessment of job satisfaction was inadequate, due to it define satisfaction as an affect but then do not treat separately the three components of satisfaction: evaluative judgments, affective job experiences and beliefs about jobs (Weiss, 2002). Applying this assessment, different studies have tested the role of affective job satisfaction among different type of professions and works. Among general workers (Rice, 2004), Norwegian farmers (Muri et al., 2020) or professors from the Kazakhstani medical universities (Uristemova et al., 2023), the predictive power of affective job satisfaction has been previously tested.

A plethora of empirical studies have connected Job Satisfaction with Emotional Intelligence, considering the mutual relationships between the two constructs. The findings supported strong mutual influences between them among different professions (Sandroto and Fransiska, 2021), specifically educators (Taliadorou and Pashiardis, 2015), and both in western societies and Asian populations (Feyerabend et al., 2018; Fida et al., 2019; Lu et al., 2021; Sabahi and Dashti, 2016; Sandroto and Fransiska, 2021; Shooshtarian et al., 2013; Wen et al., 2019; Yan et al., 2018).

### Organizational identification

Tajfel (1974) Social Identity Theory is highly relevant in Social Psychology and has exerted significant influence in recent decades, focusing on group behavior and intergroup relations. In broad terms, organizational identity refers to what employees feel, value, and think about their company, constituting a collective opinion shared by all members and characterized by the organization’s distinctive values and features. Consequently, organizational identity pertains to the social group rather than the individual (Haslam et al., 2003).

For organizational identification to occur, employees must define themselves as part of a social group distinct from the external environment, establishing behavioral norms concerning interactions with competitors and collaborators (Haslam, 2004). Organizational Identification enables individuals to embrace the view that their organization possesses unique attributes that differentiate it from others, impacting not only the organization as a whole but also every individual member, work group, department, or union, among others.

Numerous studies have demonstrated that the stronger an individual identifies with a social group, the more their attitudes and behaviors are influenced. Organizational Identification becomes a powerful determinant of commitment, performance, and organizational citizenship behaviors. Moreover, it has been shown to be a strong predictor of job satisfaction, work engagement, motivation, job performance, and citizenship behaviors, among other outcomes (Ashforth and Mael, 1989; Haslam et al., 2000; Hogg and Terry, 2000; Mael and Ashforth, 1992; Postmes et al., 2005; Riketta, 2005).

Despite numerous research studies, to the best of our knowledge, there are no existing studies that link Perceived Emotional Intelligence with Organizational Identification. Therefore, the purpose of this research is to examine the mediating role of Perceived Emotional Intelligence, encompassing emotional attention, emotional clarity, and emotional repair, in the relationship between Affective Job Satisfaction and Organizational Identification. It is expected that individuals who perceive themselves as emotionally intelligent will effectively manage their emotions and adapt better to the work environment, resulting in not only higher job satisfaction but also increased identification with the organization (Van Knippenberg and Van Schie, 2000).

### Chinese technological workers

A “technological worker” refers to an individual deeply entrenched in the realm of technology in their professional endeavors. Such individuals could be software developers, designing, writing, and testing software applications; IT specialists managing and troubleshooting information technology infrastructures; or database administrators ensuring data’s integrity, security, and availability (Dorschel, 2022). Systems analysts come into play when there is a need to design solutions for specific business problems through technology, while cybersecurity experts remain vigilant against digital threats. The digital landscape also benefits from network engineers who shape the design and management of computer networks and hardware engineers who focus on the production of computer components. Beyond the more traditional roles, the tech realm also includes research scientists delving into areas like artificial intelligence or quantum computing. Additionally, the expansive nature of technology has given rise to professions like digital artists and designers who harness technology’s capabilities for creative endeavors. In essence, a technological worker is someone whose professional life is intertwined with and reliant on the advancements and tools of modern technology (Su et al., 2022).

In the rapidly evolving tech industry of China, characterized by its dynamism, competition, and innovation, the significance of Emotional Intelligence among technological workers cannot be understated (Dheeba and Tamizharasi, 2022). The ways in which these workers perceive their emotional intelligence can have profound implications on their relationship with their employing organizations and their emotional well-being at work. Specifically, a technological worker’s Emotional Intelligence can influence their sense of alignment and connection with the company’s values and objectives. This sense of belonging or identification with the organization often translates to increased loyalty and dedication, which are especially valuable in the high-pressure environment of tech. Furthermore, those who believe they have high emotional intelligence may find themselves better equipped to navigate interpersonal complexities, manage the pressures of their roles, and adapt to the swift pace of technological change. This, in turn, can directly influence their emotional satisfaction at work. In essence, for Chinese technological workers, the nuances of how they perceive their own emotional intelligence can be pivotal in determining their emotional ties to their job and organization, with broad implications for both individual well-being and organizational outcomes.

### Mediating role of emotional intelligence between affective job satisfaction and organizational identification among Chinese technological workers

The intricate relationship between Affective Job Satisfaction and Organizational Identification is increasingly recognized as a crucial determinant of organizational success. Within this nexus, the potential mediating role of Emotional Intelligence—encompassing emotional attention, clarity, and repair—emerges as a pivotal area of investigation, particularly within the dynamic and demanding context of the Chinese technological sector. While studies like Goleman (1995) have underscored the broad impact of Emotional Intelligence on workplace outcomes, the specific mediating role of Emotional Intelligence between job satisfaction and organizational identification, especially in culturally and economically distinct settings like China, remains underexplored.

In Organizational Psychology, Affective Job Satisfaction has been linked to emotional well-being and alignment with organizational values (Cano Ibarra et al., 2023; Smith et al., 1969). Organizational Identification, as conceptualized by Ashforth and Mael (1989), reflects the degree to which employees’ identities align with their organization, influencing their commitment and performance. This intersection presents a fertile ground for examining Emotional Intelligence, especially in environments where emotional competencies are paramount.

The relevance of this research is accentuated in the context of China’s unique socio-cultural backdrop, influenced by Confucian values emphasizing harmony, collective well-being, and interpersonal relationships (Bond and Hwang, 1986; Hofstede, 1983). In such a setting, Emotional Intelligence dimensions are crucial for navigating workplace emotional landscapes, affecting both job satisfaction and organizational identification.

Moreover, the technological sector in China, characterized by rapid innovation and high-pressure environments, underscores the need for this study. The intense demand for adaptability and emotional resilience in this sector suggests a specific need to understand how Emotional Intelligence operates here. Foundational studies by Mayer et al. (2004) have laid the groundwork for understanding Emotional Intelligence’s broad impacts, but there is a gap concerning its specific mediating role in job satisfaction and organizational identification within the Chinese technological workforce.

This research aims to bridge this gap, investigating how Emotional Intelligence subdimensions mediate between job satisfaction and organizational identification. It seeks to contribute to the theoretical enrichment of Emotional Intelligence literature and offer practical insights for managing emotional dynamics in high-stress, culturally specific work environments. The anticipated findings will provide a nuanced understanding of leveraging Emotional Intelligence in organizational settings, particularly in sectors where emotional agility and interpersonal relationships are critical to employee satisfaction and identification with the organization.

Based on the aforementioned, the following hypotheses are proposed:

*H*1: Affective Job satisfaction will be directly and positively related to Organizational Identification among Chinese technological workers.

*H*2: Affective Job satisfaction will be directly and positively related to emotional attention (H2a), emotional clarity (H2b), and emotional repair (H2c) among Chinese technological workers.

*H*3: Organizational Identification will be directly and positively related to emotional attention (H3a), emotional clarity (H3b), and emotional repair (H3c) among Chinese technological workers.

*H*4: Emotional attention (H4a), emotional clarity (H4b), and emotional repair (H4c) will mediate the relationship between Affective Job satisfaction and Organizational Identification among Chinese technological workers.

## Methods

### Participants

A final sample of 392 Chinese technological workers participate (48.7% men), 44.1% of them working in a public (state-owned or partial state-owned enterprise), and the mean age was 42.73 years (S.D.: 9.7). The majority of the participants held a Bachelor’s degree (62.5%), while 11.5% had a Post-Secondary School, and 26.1% had completed a Junior Secondary or Senior Secondary School.

### Instruments

*Perceived emotional intelligence*: The Trait Meta-Mood Scale (TMMS-24) developed by Salovey et al. (2002), was used in this study. It includes 24 items, distributed into three dimensions, each consisting of eight items: (a) Emotional attention, which measures the individual’s ability to recognize and express feelings appropriately. Example of items is: *I pay close attention to my feelings*. (b) Emotional clarity, which assesses the effective and accurate understanding of one’s emotional states. Example of items is: *I am clear about my feelings*. (c) Emotional repair, which refers to the capacity to regulate emotional states. Example of items is: *I try to have positive thoughts, even when I feel bad*. The scale was presented in a 5-point Likert format, ranging from 1 (*Strongly disagree*) to 5 (*Strongly agree*). Despite a previous study cited the use of this questionnaire with Chinese samples of military students (Li et al., 2002), the full version of the scale in Chinese language has not been published. In the present study, as explained below, a back translation process has been used. In order to confirm the multidimensionality of the scale, a Confirmatory Factor Analysis is reported below.

*Affective Job satisfaction*: was measured using the BIAJS (Brief Index of Affective Job Satisfaction) developed by Thompson and Phua (2012). This scale consists of four items. An example of items is: *I really enjoy my job*. The scale was presented in a 5-point Likert format, ranging from 1 (*Strongly disagree*) to 5 (*Strongly agree*). The scale demonstrated high reliability (α = 0.92), and is a unidimensional construct in this study.

*Organizational identification*: was measured using the Organizational Identification Scale developed by Van Dick et al. (2004). This scale consists of three items. Given that there is not Chinese version of this instrument, we conducted a back translation process from English to Chinese following the procedure of previous studies with non-English speaking populations, as Pangarso et al. (2022). The construct is unidimensional. An example of items is *I identify myself as a member of the organization*. The scale was presented in a 5-point Likert format, ranging from 1 (*Strongly disagree*) to 5 (*Strongly agree*). A Cronbach’s alpha coefficient of 0.82 was obtained.

Sociodemographic data as age, gender, and educational level have been assessed.

### Procedure

In order to collect a wide sample of Chinese technological workers, a virtual sampling was conducted during the first 3 months of 2023, using a virtual snowball sampling via email. For private companies, commercial emails published on the internet and business directories were used, while for public companies, publicly available databases providing corporate emails were utilized. In an Informed consent provided to those who answered the invitation, the research objective was explained, voluntary participation was requested, and anonymity of their responses was guaranteed. The present study was approved by the (ZZUIRB) Zhengzhou University Institutional Review Board. Participants were also offered support from the Research Team for any questions that might arise. Finally, the questionnaire link, using a web-based survey tool (Qualtrics) was provided to them. The total number of participants was 435, but the surveys have been first checked and all of them that did not reach at least the 80% of the survey were eliminated. Only 90.4% without missing data remained giving us a sample of 392 for final data analysis.

### Data analyses

First, descriptive and correlation analyses were conducted. Secondly, a Confirmatory Factor Analysis has been conducted for the TTMS-24 scale, due to its multidimensionality. Subsequently, a mediation analysis was performed to examine the direct effects between Affective Job satisfaction and Organizational Identification (Hypothesis 1), as well as between the three subdimensions of Perceived Emotional Intelligence (Hypotheses 2a, 2b, 2c) and Organizational Identification. Furthermore, the indirect effects of the three subdimensions on the relationship between Affective Job satisfaction and Organizational Identification were assessed (Hypothesis 4). The JASP version 0.18.1 was employed to analyze the indirect effects, providing estimates, standard errors, and confidence intervals for multiple mediators. Bootstrapping, a non-parametric resampling procedure that does not assume normality in the sampling distribution, was utilized. Indirect effects were considered statistically significant if the 95% confidence intervals did not include zero.

## Results

Data from singular sources, like a questionnaire survey, can be influenced by common method bias (Podsakoff et al., 2003). To ensure clarity and comprehension for respondents, we undertook a back-translation process between Chinese and English, made modifications with the help of experts, and pretested the questionnaire. To counteract common method bias, we ensured respondents’ anonymity, clarified that responses were not limited to just “yes” or “no,” allowing participants to freely share their thoughts (Zhou and Long, 2004). This survey was distributed to staff members across various private and public entities in China using a web-based survey which displays the scales for the different variables along different web-pages. By this procedure we avoid the location of the items in close proximity to one another. As shown in Table 1, Organizational Identification was positively and significantly related to emotional clarity, emotional repair, and Affective Job satisfaction. There was no significant relationship between Organizational Identification and emotional attention. On the other hand, Affective Job satisfaction was positively related to emotional clarity, and emotional repair, and to emotional attention, but the latest relationship failed to show statistical significance. Finally, the relationships between the subdimensions of emotional intelligence were all positive and significant, except for the relationship between emotional repair and emotional attention.

**Table 1:** Descriptive statistics and Pearson’s matrix correlation (*N* = 392).

Variables	Mean	S.D.	1.	2.	3.	4.	5.
1. Age	42.73	9.74	1				
2. Organizational identification	3.90	0.82	0.111*	1			
3. Emotional attention	3.41	0.77	−0.066	0.048	1		
4. Emotional clarity	3.73	0.63	0.060	0.249**	0.165**	1	
5. Emotional repair	3.79	0.69	0.075	0.209**	0.090	0.396**	1
6. Affective job satisfaction	3.57	0.98	0.048	0.548**	0.098	0.253**	0.312**

*p < 0.05; **p < 0.01; ***p < 0.001.

### Confirmatory factor analysis

Data on the Emotional Intelligence scale have been submitted to Confirmatory Factor Analysis in order to test the replicability of the three factors solution. The Kaiser-Meyer-Olkin test provided good result (0.871), as well as the Bartlett’s Sphericity test (Chi squared = 4876.74; d.f.: 276; *p <* 0.001). The factor loadings for each item are displayed in Table 2, being adequate in general. Item 23, from the Emotional reparation subscale showed a low factor loading, despite its statistical significance.

**Table 2:** Confirmatory factor analysis for the TTMS-24 scale.

Factor	Item	Estimate	*z*-value	*p*	Reliability
Emotional attention					Omega = 0.881 Alpha = 0.882
	Emotional attention 1	0.567	11.276	<0.001	
	Emotional attention 2	0.690	14.507	<0.001	
	Emotional attention 3	0.763	15.253	<0.001	
	Emotional attention 4	0.668	14.228	<0.001	
	Emotional attention 5	0.569	11.273	<0.001	
	Emotional attention 6	0.851	16.631	<0.001	
	Emotional attention 7	0.863	17.252	<0.001	
	Emotional attention 8	0.845	17.242	<0.001	
Emotional clarity					Omega = 0.865 Alpha = 0.861
	Emotional clarity 9	0.667	14.598	<0.001	
	Emotional clarity 10	0.746	18.859	<0.001	
	Emotional clarity 11	0.645	17.115	<0.001	
	Emotional clarity 12	0.419	10.972	<0.001	
	Emotional clarity 13	0.408	11.492	<0.001	
	Emotional clarity 14	0.676	13.682	<0.001	
	Emotional clarity 15	0.571	12.517	<0.001	
	Emotional clarity 16	0.592	14.207	<0.001	
Emotional repair					Omega = 0.889 Alpha = 0.880
	Emotional repair 17	0.813	17.892	<0.001	
	Emotional repair 18	0.849	20.116	<0.001	
	Emotional repair 19	0.834	17.093	<0.001	
	Emotional repair 20	0.793	19.074	<0.001	
	Emotional repair 21	0.553	12.134	<0.001	
	Emotional repair 22	0.516	12.618	<0.001	
	Emotional repair 23	0.236	7.498	<0.001	
	Emotional repair 24	0.633	15.481	<0.001	

The reliability of the subscales was adequate, as displayed in this table.

### Mediation analysis

The total effect model explains more than 30% of the variance in Organizational Identification, while the percentage of explained variance in Emotional attention was 0.010, in Emotional clarity 0.064 and in Emotional repair was 0.097. As shown in Table 3, the analyses revealed a significant total effect between Affective Job satisfaction and Organizational Identification, while the direct effect is also significant. The direct path coefficients of the dimensions of Emotional Intelligence on Organizational Identification were only significant for emotional clarity, while they were not significant for emotional attention or emotional repair.

**Table 3:** Parameter estimates for the mediational analyses.

Total effects							95% CI	
			Estimate	Std. Error	*z*-value	*p*	Lower	Upper
Affective Job satisfaction	→	OI	0.555	0.043	12.964	<0.001	0.471	0.639
Direct effects							95% CI	
			Estimate	Std. Error	z-value	*p*	Lower	Upper
Affective Job satisfaction	→	OI	0.526	0.045	11.638	< 0.001	0.438	0.615
Indirect effects							95% CI	
			Estimate	Std. Error	*z*-value	*p*	Lower	Upper
Affective Job satisfaction	→	OI	0.029	0.017	1.740	0.082	−0.004	0.062

Delta method standard errors, normal theory confidence intervals, ML estimator. OI, Organizational Identification.

**Figure 1:**
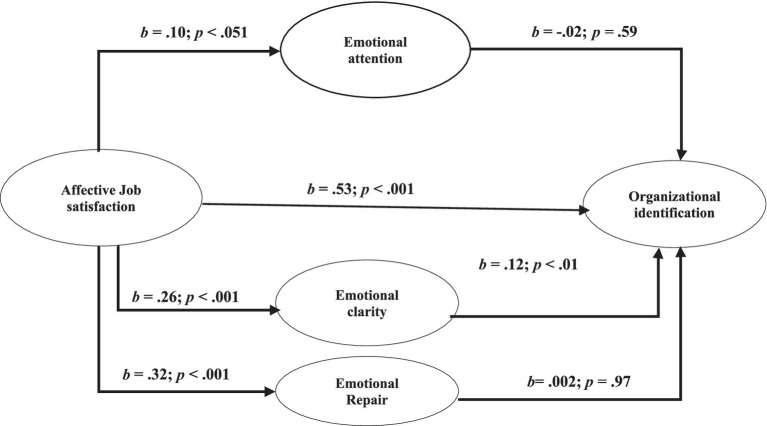
Results of the mediational model.

In the analysis of the overall model, the indirect effect of emotional clarity on Organizational Identification was the only statistically significant one, as shown Table 4. In contrast, emotional repair showed a practically irrelevant indirect effect on Organizational Identification, as did emotional attention, which is negative. In summary, the results show uneven support for the proposed hypotheses. Figure 1 graphically represents the main results.

**Table 4:** Indirect effects.

					Estimate	Std. Error	*z*-value	*p*	Lower	Upper
Affective job satisfaction	→	Emotional attention	→	OI	−0.002	0.004	−0.517	0.605	−0.011	0.006
Affective job satisfaction	→	Emotional clarity	→	OI	0.031	0.013	2.312	0.021	0.005	0.057
Affective job satisfaction	→	Emotional repair	→	OI	0.00049	0.015	0.034	0.973	−0.029	0.030

## Discussion

Central to the novelty of our study is the unique exploration of the interplay between Perceived Emotional Intelligence and job satisfaction within the context of the Chinese technological sector. This focus is particularly innovative as it traverses two relatively underexplored domains: the specific nuances of Emotional Intelligence in a rapidly evolving technological landscape and the cultural dimensions inherent in the Chinese workforce. Our findings not only enrich the existing understanding of Emotional Intelligence in the workplace but also shed new light on how these dynamics manifest in a critical sector within one of the world’s largest economies. Moreover, by dissecting the subdimensions of Emotional Intelligence—emotional attention, clarity, and repair—our research provides a more nuanced understanding of how these individual aspects contribute to, or diverge from, the overall relationship between job satisfaction and organizational identification.

Furthermore, this research disrupts the traditional narrative by offering fresh insights into the directional influence between job satisfaction and Perceived Emotional Intelligence. The significant correlation we identified between emotional clarity and organizational identification, in particular, underscores the pivotal role of this dimension in a technological work environment that is both highly demanding and rapidly changing. This aspect of our study not only contributes to the broader discourse on Emotional Intelligence in the workplace but also presents a compelling case for the need to tailor Emotional Intelligence interventions to specific subdimensions, based on the unique requirements of different organizational contexts. By doing so, our study paves the way for more effective, context-sensitive strategies in human resource management and organizational development, especially in sectors where emotional competencies are critical to both individual and organizational success.

The aim of this research was to investigate the mediating role of the three subdimensions of Perceived Emotional Intelligence—emotional attention, emotional clarity, and emotional repair—in the relationship between organizational identification and job satisfaction in a sample of technological workers from China. Overall, the findings suggest that emotional clarity significantly contributes to addressing organizational challenges and positively influences organizational identification. In contrast, neither emotional attention nor emotional repair appear pivotal in successfully addressing the varied challenges and demands faced by both public and private organizations today.

Initially, results indicate a direct and positive association between affective job satisfaction and organizational identification (H1). This is consistent with prior studies, such as the one involving 175 permanent faculty members from a private university in Karachi, which demonstrated a direct positive relationship between job satisfaction and organizational identification (Kazmi and Javaid, 2022). The findings also align, in part, with a study involving 352 technological workers from a top Indian IT organization. In this research, Bharadwaj et al. (2022) observed that an enhanced organizational identity among satisfied employees curtails the intention to leave among IT professionals.

Furthermore, the results confirm that satisfied workers score high in both emotional clarity and emotional repair (H2b and H2c), but no direct positive relationship with emotional attention was observed (H2a). Our findings somewhat align with the relationship between facets of Emotional Intelligence and Job Satisfaction observed among banking sector employees in Pakistan (Fida et al., 2019). However, it’s worth noting that a different set of Emotional Intelligence dimensions was evaluated in that study. Similarly, Wen et al. (2019) determined a robust direct relationship between Emotional Intelligence and Job Satisfaction, with other variables partially mediating this link among hotel industry employees in China. Drawing from a sample of 356 female clinical nurses across two Chinese hospitals, Yan et al. (2018) also identified a positive and notable association between Emotional Intelligence and Job Satisfaction.

Diving deeper, among public service workers in South Korea, Lee (2021) discovered that only the self-regulation aspect of Emotional Intelligence acted as a mediator between emotional labor and Job Satisfaction. This suggests that not all dimensions of Emotional Intelligence function uniformly. Echoing this sentiment, Lu et al. (2021)found that solely the self-regulation facet of Emotional Intelligence had a significant and positive influence on Job Satisfaction among Chinese public employees.

This series of studies, particularly those focusing on Chinese employees, are grounded in the recognition of Confucianism’s influence on understanding Chinese culture. Central to Confucianism is the value of benevolence, which often directs Chinese individuals toward seeking social harmony and cultivating peaceful relations with others (Lee and Ding, 2008; Li, 2013). This cultural backdrop plays a significant role in shaping workplace behaviors and attitudes in China (Hofstede, 1984; Yau, 1988).

Confucianism, with its emphasis on values such as harmony, respect for authority, and the importance of relationships (guanxi), profoundly influences the interpersonal dynamics within Chinese organizations (Lee and Ding, 2023). For instance, the Confucian concept of ‘Harmony but not Sameness’ (和而不同) encourages a balanced approach to workplace interactions, promoting cooperation and conflict avoidance (Li, 2013).

Consequently, employees with elevated levels of emotional intelligence are better equipped to discern emotions and navigate emotional challenges in this context, ensuring they meet job demands proficiently (Yang and Bond, 1980). As a result, those who feel at ease in their professional settings are likely to leverage their emotional competencies more effectively. Studies have shown that in cultures where Confucian values are prevalent, emotional intelligence is particularly important in facilitating effective workplace relationships and outcomes (Han and Altman, 2010).

By understanding these cultural nuances, it becomes evident that the role of Emotional Intelligence in the workplace, especially in a Chinese context, extends beyond personal competency to encompass a broader cultural and social framework, deeply rooted in Confucian philosophy.

Although most empirical studies have examined the inverse relationship, namely the influence of PEI on job satisfaction, it is crucial to acknowledge two issues. First, the vast majority of published studies are correlational, so the potential influence of job satisfaction on PEI cannot be entirely ruled out. Second, these studies typically focus on cognitive job satisfaction, or on job satisfaction generally but assessed cognitively. That is, the instruments used measure satisfaction in terms of how current working conditions match a previously established standard. Both these caveats in prior research suggest that data regarding the direction of influence between PEI and Affective Job Satisfaction should be interpreted with caution. Additionally, these results imply that the subdimensions of emotional clarity and repair are closely linked to adequate job satisfaction as they enable individuals to manage moods and adapt better to work-related challenges (Diefendorff et al., 2008).

Regarding the relationship between emotional attention and repair and organizational identification, our findings do not confirm a direct link for H3a and H3c. However, there is a statistically significant direct correlation between emotional clarity (H3b) and increased organizational identification. This highlights the pivotal role of emotional clarity competencies in fostering enhanced organizational ties. A plausible inference is that greater emotional clarity might lead to better interpersonal relationships with colleagues, supervisors, clients, and service recipients. This can subsequently aid adaptation in the fast-paced, evolving environment typical of technological workers, especially when pressed to meet performance objectives swiftly. Consistently, past research indicates a significant positive correlation between Emotional Intelligence and Organizational Identification, as evidenced by studies such as Azizi et al. (2018). Zehir et al. (2019) further supported this connection in their study involving 314 employees across various organizations.

Finally, regarding hypothesis 4, neither emotional attention (H4a) nor emotional repair (H4c) mediates the relationship between affective job satisfaction and organizational identification. Hence, merely being aware of and identifying or repairing emotions does not enhance organizational identification. Conversely, emotional clarity (H4b) might promote more efficient processing of information related to emotions and feelings in the work environment. This likely leads to improved emotional adjustment, consequently positively influencing their identification with the organization.

### Limitations of the present study and recommendations for interventions in organizations with technological workers in China

Our study predominantly focused on technological workers in China, and as such, the findings might not be easily generalizable to non-technological sectors or to workers in different cultural contexts. Another inherent limitation was the study’s cross-sectional design. While it can identify associations, it does not pinpoint causal relationships. A more longitudinal approach in future research could shed light on causality and the temporal sequence of the examined variables. Additionally, we relied heavily on self-report surveys. These can introduce elements of social desirability biases and might not comprehensively capture the intricate dynamics of emotions. Furthermore, there is a possibility that other influential factors, such as managerial styles, organizational culture, or external socio-economic conditions, which were not factored into this study, might play a role in the observed outcomes.

In light of the significance of emotional clarity, organizations are advised to invest in emotional intelligence training programs that specifically target this subdimension. By enhancing workers’ ability to understand their emotions, it is plausible that both their performance and job satisfaction could see marked improvement. Moreover, when tailoring interventions for technological workers in China, it is crucial to integrate the unique cultural values, norms, and expectations that mold their work-related perceptions and experiences (Liang et al., 2022). Establishing mentorship programs can also be beneficial. Through these programs, seasoned workers can offer guidance to newer employees, helping them navigate organizational challenges while utilizing their emotional intelligence skills. Additionally, fostering an open organizational culture, where employees can freely express their feelings, challenges, and concerns, can mitigate potential misunderstandings stemming from cultural or role-based expectations. Given the inherently dynamic and often high-pressure nature of the technological sector, it is essential for organizations to prioritize the well-being of their employees. This can manifest as mental health initiatives, be it regular check-ins, relaxation zones, or even offering counseling services. Lastly, regular feedback mechanisms should be in place. By providing consistent feedback, employees can achieve clarity on their roles, performance, and any emotional challenges they face, engendering a culture of continuous growth and support.

Incorporating these recommendations and acknowledging the discussed limitations can guide both future research and organizational interventions to cater more effectively to the distinct needs of technological workers in China.

## Conclusion

The present study provides valuable insights into the intricate relationship between Perceived Emotional Intelligence and its role in mediating organizational identification and job satisfaction among technological workers in China. Notably, our research underscores the pivotal role of emotional clarity in shaping these relationships, suggesting its potential as a key intervention area for organizations aiming to bolster worker satisfaction and identification. Moreover, while some aspects of Perceived Emotional Intelligence showed limited impact, the overall findings emphasize the importance of emotional intelligence in the contemporary workplace. In doing so, this research not only expands the existing body of knowledge but also offers actionable pathways for organizations in the technological sector, particularly within the unique cultural context of China, to enhance both employee well-being and organizational cohesion.

## Data Availability

The raw data supporting the conclusions of this article will be made available by the authors, without undue reservation.
